# The Efficacy of a Long-Acting Injectable Selenium Preparation Administered to Pregnant Ewes and Lambs

**DOI:** 10.3390/ani11041076

**Published:** 2021-04-09

**Authors:** Stanisław Milewski, Przemysław Sobiech, Justyna Błażejak-Grabowska, Roman Wójcik, Katarzyna Żarczyńska, Jan Miciński, Katarzyna Ząbek

**Affiliations:** 1 Department of Sheep and Goat Breeding, Faculty of Animal Bioengineering, University of Warmia and Mazury in Olsztyn, Oczapowskiego 5, 10-719 Olsztyn, Poland; stanmil@uwm.edu.pl (S.M.); justyna.blazejak@uwm.edu.pl (J.B.-G.); micinsk@uwm.edu.pl (J.M.); 2 Department of Internal Diseases, Faculty of Veterinary Medicine, University of Warmia and Mazury in Olsztyn, Oczapowskiego 14, 10-719 Olsztyn, Poland; psobiech@uwm.edu.pl (P.S.); katarzyna.zarczynska@uwm.edu.pl (K.Ż.); 3 Department of Microbiology and Clinical Immunology, Faculty of Veterinary Medicine, University of Warmia and Mazury in Olsztyn, Oczapowskiego 13, 10-719 Olsztyn, Poland; brandy@uwm.edu.pl

**Keywords:** barium selenate, sheep, immunity, performance

## Abstract

**Simple Summary:**

Lack of selenium (Se) is a global problem that leads to increased exposure of animals to various diseases, as well as a reduction in productive and reproductive performance. Mineral supplementation can improve the immune system and could therefore be of assistance for lambs. We studied the effects of injectable supplementation with selenium. Supplementation of the Se lead to improved immune status, blood parameters, body weights, and average daily gain of lambs.

**Abstract:**

The aim of this study was to evaluate the effects of a long-acting selenium (Se) preparation administered to sheep. The experiment was conducted on 30 dams and 36 lambs divided into three equal groups of 10 dams and 12 lambs each: Control—C, and two experimental groups—E (Se administered to pregnant ewes) and EI (Se administered directly to lambs after the colostral period). The Se preparation (Barium Selenate Injection, BVP Animal Care, 50 mg/mL) was administered by injection at 1 mL/50 kg (1 mg Se/kg) body weight (BW) to group E ewes in the third month of pregnancy (between 70 and 90 days) and to group EI lambs between 4 and 7 days of age. The following parameters were determined: Se concentration in the blood of ewes, milk yield, milk composition, Se concentration in milk; hematological, biochemical, and immunological parameters and Se concentration in the blood of lambs; growth rate and in vivo measurements of lean meat and fat content in lambs. Barium selenate significantly improved the Se status of dams and lambs, regardless of whether it was administered to pregnant ewes or directly to lambs in the first week of their life. The milk of ewes receiving the Se preparation was characterized by higher concentrations of fat and dry matter. The Se preparation induced significant changes in immunological parameters, thus enhancing defense mechanisms in lambs. The Se preparation exerted more stimulatory effects on humoral and cellular immune responses when administered directly to lambs after the colostral period (group EI) than to pregnant ewes (group E). The results of this study indicate that the long-acting Se preparation delivers benefits to sheep by boosting their immunity and, therefore, improving performance.

## 1. Introduction

Selenium (Se) is a trace element that is required for healthy body functioning, and Se deficiency can compromise the health status of animals and, consequently, decrease their components and biological availability [1,2]. Se is common on the earth’s surface, but its concentration and distribution are not uniform. There are areas with soils either poor or rich in this element. Most soils in the world have low content of this element. Se deficiency is more of a problem geographically than is selenium toxicity. The selenium content of forages varies with the type of feed, the type of soil, and the region [2]. Se levels are low in soil and in green forage that is consumed by sheep in the basal diet. In ruminants, this micromineral is relatively poorly absorbed from feed because rumen microbes partially reduce much of dietary inorganic selenium to unabsorbable elemental or inorganic selenide forms [3].

The absorption of selenium from the given food differs significantly in mono and polygastric animals. In the former it reaches 80%, while in ruminants it does not exceed 51% [4]. Calves, lambs, and goatlings use significantly more of this element than adults—it is related to the still undeveloped rumen function. The main rumen microorganisms that can incorporate selenium into their structures are bacteria belonging to the species: *Butyvibrio fibrisolvens*, *Prevotella ruminicola*, *Selenomonas ruminantium*, as well as *Streptococus* sp. *and Lactobacillus* sp. [5]. Pure cultures of *Selenomonas ruminantium* and *Butyvibrio fibrisolvens* incorporate selenium into selenoamino acids, while *Prevotella ruminicola* metabolizes selenium compounds into an elemental form—inaccessible to a higher organisms [6]. Studies performed on sheep have shown [7] that lower selenium absorption in animals fed green fodder is associated with the presence of the latter microorganism, while in the high-energy diet (concentrated feed) in the rumen, *Selenomonas ruminantium* bacteria predominate, increasing the bioavailability of the collected element.

Therefore, livestock diets have to be supplemented with Se. In sheep, Se is generally administered via the parenteral route. The most commonly used is sodium selenate and vitamin E preparations with a period of action not exceeding a few weeks, and the treatment has to be repeated. Long-acting preparations that release Se over a period of several months appear to be a more effective solution. Barium selenate is one of such formulations. However, the effectiveness of barium selenate supplements remains insufficiently investigated. In a study by Annett et al. [8], barium selenate significantly improved the Se status of lambs. Ceballos et al. [9] demonstrated that barium selenate administered one month before calving effectively prevented mastitis in dairy cows.

The aim of this study was to determine the efficacy of a single injectable dose of barium selenate administered to pregnant ewes or to lambs in the first week of their life. The research hypothesis states that barium selenate increases Se concentrations in the blood and milk of ewes, increases milk yield, improves milk composition, enhances the health status of lambs, and improves meat performance.

## 2. Materials & Methods

The experiment was approved by the Local Ethics Committee for Animal Experimentation of the University of Warmia and Mazury in Olsztyn (decision No. 68/2014). The study was conducted on a breeding herd of Pomeranian sheep. The study was conducted in Baldram (Poland), located at 53°45′ North latitude, 18°56′ East longitude. Thirty ewes aged 3–4 years were divided into two analogue groups: C—control (15 ewes) and E—experimental (15 ewes), based on age and body weights (BW). The groups were established at the end of the third month of pregnancy (between 70 and 90 days—this moment is understood as day 0), as confirmed by an ultrasound examination performed with the Mindray DP50 scanner with an abdominal probe in the 3.5–5 MHz frequency range. Experimental group ewes were subcutaneously (skin fold neck) injected with a long-acting Se preparation (Barium Selenate Injection, BVP Animal Care, Ireland, 50 mg of Se in 1 mL) at 1 mL/50 kg body weight, which gives dosage 1 mg Se/1 kg BW of ewes. Based on birth result, 10 mothers and their 12 lambs (including 8 singletons and 4 twins, with equal proportions of each sex) were selected for further research from each of these groups (C and E). During lambing, a second experimental group (EI) composed of 10 dams and 12 lambs from other animals kept in the herd was created. Group EI animals were identical to the remaining groups in terms of the BW and age of dams, the BW of lambs, the number of singletons and twins, and the sex ratio. Third group EI (12 lambs) were injected with barium selenate at a dose of 1 mL/50 kg BW between 4 and 7 days of age (day 0), which gives dosage 1 mg Se/1 kg BW of lambs. The lambs were kept with their dams until 100 days of age. Dam diets had the following composition (kg/animal/day): Meadow hay—1.0; barley straw—0.5; dried beet pulp—0.5; CJ concentrate—0.3 during pregnancy and 0.6 during lactation. Beginning at 11 days of age, suckling lambs had ad libitum access to meadow hay and the CJ concentrate. The chemical composition of the diets is presented in Table 1. The CJ^®^ concentrate had the following composition: Ground barley (40%), ground wheat (37.5%), ground maize (10%), soybean meal (10%), mineral premix (2%), fodder chalk (0.2%), dicalcium phosphate (0.2%), and salt fodder (0.1%). Animals all groups had unrestricted access to Multi-Lisal salt licks, which contains: Nacl—94%, water—insoluble substances—max. 4%, Mg 2000 mg/kg, Co—18 mg/kg, Zn (zinc)—810 mg/kg, Mn—830 mg/kg, I (Iodine)—100 mg/kg, Se (selenium)—10 mg/kg. Due to the fact that in the whole herd the feeding program included constant access to licks for all animals, therefore, the supply of selenium from the licks was assumed in the same dosage for both the control and experimental groups.

Dried beet pulp was incorporated into lamb diets on day 31, and the composition of lamb diets was normalized. The quantity of each dietary ingredient in the daily ration of lambs was increased by 0.05 kg every 10 days, beginning with 0.15 kg of hay, 0.15 kg of the CJ concentrate, and 0.05 kg of dried beet pulp between days 31 and 40 of age.

Blood samples were collected from the jugular vein. Hematological parameters: Red blood cell counts (RBC), white blood cell counts (WBC), hemoglobin (HBG), hematocrit (HCT), mean corpuscular volume (MCV), mean corpuscular hemoglobin (MCH), mean corpuscular hemoglobin concentration (MCHC), platelet counts (PLT), and mean platelet volume (MPV) were determined in full blood, using the Vet Animal Blood Counter 18 (Horiba ABX SAS). Blood samples for biochemical tests were collected into polyethylene tubes containing clot activating beads. Samples for biochemical analyses were centrifuged immediately after collection (10 min, 3000 rpm). The obtained serum was stored in individual test tubes at −18 °C until further analysis. Biochemical analyses of the serum were performed within 4 h after collection. The following biochemical parameters were determined: Glucose (GLU) and total protein (TP) levels; the activity of aspartate transaminase (AST), alkaline phosphatase (ALP), lactate dehydrogenase (LDH), gamma-glutamyl transpeptidase (GGT); and the concentrations of cholesterol (Chol), triglycerides (TG), and Se. The following analytical methods were used: Glucose concentration was determined with the use of glucose oxidase [10], total protein was determined by the biuret method [11], cholesterol—by the colorimetric method with cholesterol esterase and cholesterol oxidase [12], triglycerides—by the enzymatic method with glycerophosphate oxidase [13], AST activity—by the kinetic method [14], LDH activity—by the kinetic method with Cormay reagents [15], GGT activity—by the kinetic method with l-glutamyl-3-carboxy-4-nitroanilide [16], ALP activity—by the kinetic method [17]. All determinations were performed using an ACCENT 200 automated chemistry analyzer (Cormay) and commercial Cormay diagnostic kits. Serum Se levels were determined in triplicate by graphite furnace atomic absorption spectrometry in LABOKLIN Laboratory for Clinical Diagnostics GmbH & Co. KG (Bad Kissingen, Germany).

The following humoral immunity parameters were determined: Lysozyme and ceruloplasmin activity and gamma globulin concentrations. Cellular immunity mechanisms were analyzed based on specific and non-specific immunity parameters: Respiratory burst activity (RBA) and potential killing activity (PKA) of phagocytes, and the proliferative response of mitogen-stimulated T-cells and B-cells. Lysozyme activity in the blood plasma was determined by the method described by Siwicki and Anderson [18], and ceruloplasmin activity—by the method proposed by Siwicki and Studnicka [19]. The serum concentrations of gamma globulins were determined by the micromethod proposed by Lowry and modified by Siwicki and Anderson [18]. Respiratory burst activity, i.e., the metabolic activity of phagocytes stimulated with phorbol myristate acetate (PMA), was measured by spectrophotometry (OD 620 nm). The PKA of polymorphonuclear and mononuclear phagocytes was determined by spectrophotometry (OD 620 nm). The proliferative response of T-cells stimulated with concavalin A (ConA) and B-cells stimulated with lipopolysaccharide (LPS) was determined by MTT-based spectrophotometry.

Selenium concentration in milk and diets were determined by inductively coupled plasma mass spectrometry (ICP-MS) in the J.S. Hamilton Poland laboratory.

Daily milk production was determined based on the results of the morning milking test performed 12 h after the previous milking. The animals were administered 2.5 U of oxytocin (Oxytocinum Biowet, Puławy) approximately 2 min before milking to stimulate the contraction of myoepithelial cells surrounding the mammary alveoli, and lactiferous ducts. The right half of the udder was milked manually, whereas the other half was simultaneously sucked by a lamb. The quantity of collected milk was multiplied by 4 to obtain the daily milk yield according to the method described by Ząbek et al. 2014 [20]. Milk was analyzed to determine the percentage content of dry matter, fat, protein and lactose, and somatic cell counts (SCC) per ml. The analyses were performed in the Combi Foss 6000 system.

The height, width, and cross-sectional area of the *musculus longissimus dorsi* (m.l.d.) and fat thickness over the loin eye were determined with the Mindray DP50 ultrasound scanner with a 5 MHz linear probe. The measurements were conducted in vivo at the level of the last thoracic vertebra [21].

The results were processed statistically, and the significance of differences between groups was verified by Duncan’s test. Blood indices and Se concentration were analyzed in a static and dynamic system relevant to day 0. Data were processed in the Statistica 13.1 program. The experiments were carried out in accordance with the established standards for animal experimentation.

Differences across treatment groups were estimated with the use of the below formula:

$$Y_{ij}=\;\mu +\;\alpha_{i}+e_{ij}$$

where: *Y_ij_*—dependent variable; *µ*—overall mean; *α_i_*—fixed effect of treatment; *e_ij_*—random residual error.

Blood indices and serum Se concentration were evaluated by repeated measures with the following formula:

$$Y_{ijk}=\;\mu +\;\alpha +\;\beta_{j}+T_{i}*t_{j}+e_{ij}$$

where: *Y_ijk_*—dependent variable; *µ*—overall mean; *α_i_*—fixed effect of treatment; *β_j_*—effect of sampling date (days 0, 28, 70 or 100); T_i_*t_j_—fixed effect of treatment x time interaction, *e_ij_*—random residual error.

## 3. Results

**Figure 1:**
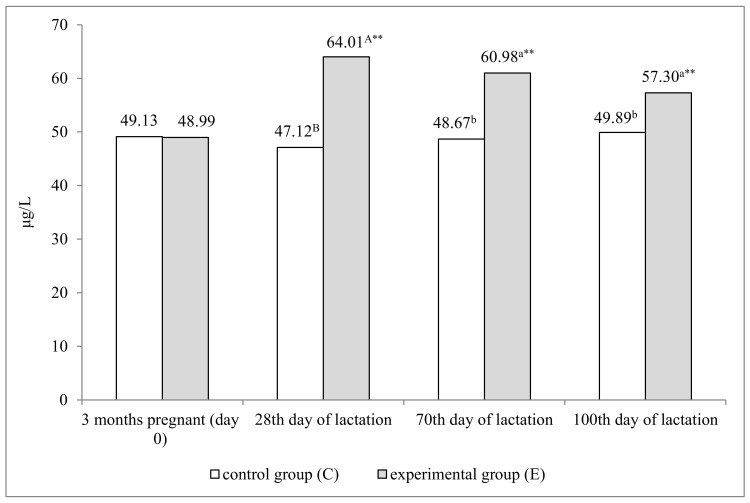
Serum Se concentration in dams from control group C and experimental group E (μg/L) a, b—*p* ≤ 0.05; A, B—*p* ≤ 0.01. ** *p* ≤ 0.01—significant difference relative to day 0 (3 months pregnant).

The Se preparation increased serum Se levels in dams (group E) (Figure 1). Dams had a similar concentration of Se in the blood on day 0—in the third month of pregnancy. Serum Se concentration was significantly higher in the experimental group than in the control group on successive days of lactation. This parameter was higher by 16.89 μg/L (*p* ≤ 0.01) on day 28, and 12.31 and 7.41 μg/L (*p* ≤ 0.01) on days 70 and 100, respectively. In these group of dams, serum Se concentration was significantly higher on every successive sampling date relative to day 0 (*p* ≤ 0.01). This parameter remained at a stable level in the control group.

Milk yield, milk quality, and Se concentration in milk are presented in Table 2. The administered preparation did not affect average daily milk yields. An analysis of the proximate chemical composition of milk revealed that on lactation day 70, fat content and, consequently, dry matter concentration were highest in group E ewes that received the Se preparation during pregnancy. The differences in both parameters were significant (*p* ≤ 0.05) relative to group C. The tested preparation had no significant effect on SCC in milk. There was no statistically significant difference in Se concentration between the milk of group E dams and milk of dams from remaining group.

The hematological parameters determined in the blood of lambs are presented in Table 3. A morphological analysis of lamb peripheral blood did not reveal significant differences in leukocyte or erythrocyte counts across groups during the entire experiment. A similar trend was noted in hemoglobin concentration. The analyzed parameters were within the reference ranges for the species. The remaining hematological parameters (MCV, HCT, MCHC, PLT, and MPV) were also similar and within the norm in all lamb groups.

The biochemical parameters determined in the blood of lambs are presented in Table 4. Serum glucose levels in groups E and EI were higher than in the control group. This trend was maintained during the entire experiment, but significant differences were not determined in the static or the dynamic approach. Total protein concentration in control group lambs ranged from 47.12 g/L to 48.91 g/L during the experiment. Groups E and EI differed in total protein concentration on successive sampling dates. On day 0, total protein was higher in group E (57.34 g/L) than in group C (48.22 g/L) and group EI (48.21 g/L). On the remaining sampling dates, total protein concentration was highest in group EI. However, the observed differences were not statistically significant. In all groups, AST activity fluctuated during the study, but the noted differences were not significant. The activities of ALP, LDH, and GGT were similar in all groups.

Triglyceride levels were similar in all lambs on the first three sampling dates, but they were significantly lower in groups E and EI on day 100 (*p* ≤ 0.05). In contrast, cholesterol concentration remained similar in all lambs during the experiment.

An analysis of humoral defense mechanisms in lambs (Table 5) revealed that the Se preparation significantly increased the values of all evaluated parameters. Lysozyme activity was highest in lambs that were directly administered the Se preparation (group EI), and significant differences relative to the remaining groups were observed on days 28, 70, and 100. In group EI lambs, lysozyme activity increased significantly (*p* ≤ 0.01) on days 70 and 100, relative to day 0. In group E lambs, lysozyme activity was higher than in group C on days 70 and 100, but the noted differences were not significant. Lysozyme activity in group E lambs was higher on day 100 relative to day 0. Similar trends were observed in ceruloplasmin activity and gamma globulin levels. These parameters were significantly higher in group EI than in the remaining groups. The observed differences were significant at *p* ≤ 0.05 on day 28 and at *p* ≤ 0.01 on days 70 and 100. A dynamic analysis revealed that both parameters increased significantly with lambs’ age, and that relative to day 0, the observed increase was significant at *p* ≤ 0.05 on day 28 and at *p* ≤ 0.01 on the remaining sampling dates.

Similar trends were noted in cellular immunity parameters (Table 5). Both non-specific (RBA and PKA of phagocytes) and specific immunity parameters (MTT-ConA and MTT-LPS) were higher in experimental lambs. Group EI lambs were characterized by highly significantly higher (*p* ≤ 0.01) values of RBA on days 70 and 100, and PKA on day 100. On days 28 and 70, PKA was significantly higher (*p* ≤ 0.05) in group EI than in the remaining groups. An analysis of the rate of changes in these parameters demonstrated that RBA increased significantly in group E on day 100 relative to day 0. Potential killing activity increased significantly on days 28 and 100 in group E lambs, and on days 70 (*p* ≤ 0.05) and 100 (*p* ≤ 0.01) in group EI lambs. Specific cellular immunity parameters (MTT-ConA and MTT-LPS) also differed between groups. On days 28 and 100, MTT-ConA was significantly higher in group EI than in the remaining groups. A dynamic analysis revealed a highly significant increase in this parameter on successive days of the experiment relative to day 0 in both groups of lambs. The value of MTT-LPS was significantly higher on day 28 and highly significantly higher on day 70 in group EI than in the remaining groups. Relative to day 0, MTT-LPS increased significantly on day 70 in groups C and E, and it increased significantly on days 28 (*p* ≤ 0.05) and 70 (*p* ≤ 0.01) in group EI.

**Figure 2:**
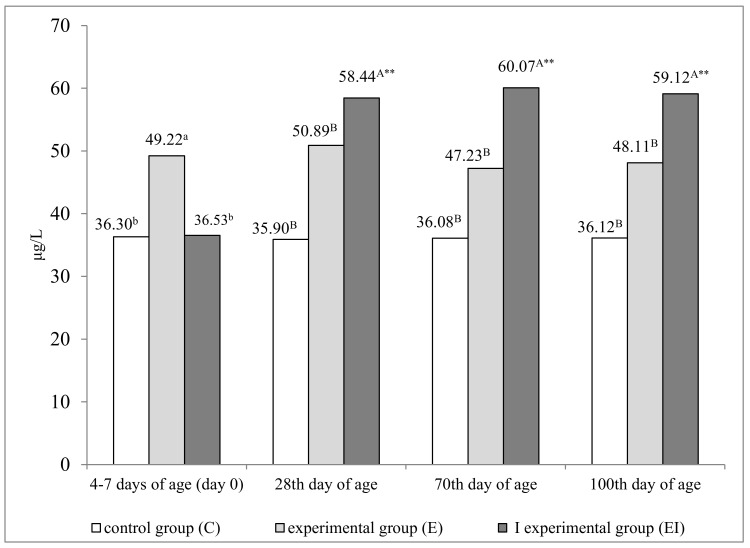
Serum selenium concentration in lambs from control group C, experimental group E, and I experimental group EI (μg/L). a, b—*p* ≤ 0.05; A, B—*p* ≤ 0.01. ** *p* ≤ 0.01—significant difference relative to day 0.

The Se preparation induced significant changes in the Se status of lambs (Figure 2), regardless of whether it was administered to pregnant ewes (group E) or directly to lambs in the first week of their life (group EI). Serum Se concentration was significantly higher (*p* ≤ 0.05) in group E lambs than in groups C and EI already on day 0. In comparison with the control group, group E lambs were also characterized by higher serum Se concentration on successive sampling dates, but the observed differences were not significant. On days 28, 70, and 100, serum Se levels were significantly highest (*p* ≤ 0.01) in group EI lambs relative to the remaining groups. This parameter was highly significantly higher on all sampling dates relative to day 0.

The growth rate and the results of in vivo measurements performed in lambs are presented in Table 6. At 100 days of age, the average BW of group E and EI lambs was higher by 1.13 kg and 3.03 kg, respectively, relative to control group lambs. However, significant differences were observed only between group EI lambs and control group animals (*p* ≤ 0.05). The above can be attributed to significantly higher ADG in group EI during the entire rearing period. In group EI, ADG was determined at 269.71 g, and it was 12.79% higher than in group C (*p* ≤ 0.05). The Se preparation also significantly influenced muscle growth in lambs (Table 6). At 100 days of age, all m.l.d parameters were significantly highest (*p* ≤ 0.01) in group EI. The width and cross-sectional area of m.l.d were significantly higher in group E than in group C lambs (*p* ≤ 0.05). Fat thickness over the loin eye was similar in all groups.

## 4. Discussion

### 4.1. Selenium Concentration in the Serum and Milk

This study demonstrated that the long-acting Se preparation increased serum Se concentration in dams, which corroborates the findings of Muñoz et al. [22]. Similar results were reported by other authors who investigated short-acting Se preparations in sheep [23,24]. The administration of Se to pregnant ewes enhanced the Se status of lambs. These observations were confirmed by other researchers who found that Se supplementation during pregnancy prevents Se deficiency in the offspring [25]. However, Barbé et al. [26] observed that the Se content of milk is determined mainly by the availability of Se in cow diets. Selenium was not detected in milk or the serum of cows administered a low dose of the Se supplement (0.1 µg/g), which suggests that a low Se dose is utilized mainly to meet the animals’ physiological needs. Serum and milk Se levels increased only in response to supplemental Se doses of 0.2 and 0.3 µg/g. In ruminants, Se is transferred more effectively through the placenta than through milk. In general, Se concentration is 3- to 5-fold higher in plasma than in milk [2]. According to Pehrson et al. [27], Se is not effectively transferred to milk; therefore, it does not guarantee healthy Se status in the offspring. In the present study, Se supplementation during pregnancy tended to increase the Se content of milk, and similar observations were made by other authors [28,29,30,31].

Literature data are quite divergent in determining the appropriate concentration of Se in the serum or blood content, therefore it is difficult to unequivocally assess the status (deficient, marginal or non-deficient) in ewes and lambs. Grace [32] reports that serum Se concentrations of <41 μg/L suggest a deficiency of this trace element 41–79 μg/L is a marginal level, and >79 μg/L is considered the physiological level. However, according to Ghany-Hefnawy [33] the optimal concentration of Se in the blood serum of ewes is higher and should be in between 120–150 μg/L.

### 4.2. Milk Yield and Composition

In the current study, Se supplementation had no effect on milk yield or the lactose and protein content of milk, and similar results were reported in cattle by Ferreira and Petzer [34]. According to Juniper et al. [35], Heard et al. [36], and Paschoal [37], organic Se does not affect the percentage content of protein or lactose in milk. Barium selenate injections administered before mating improved milk performance in goats by decreasing SCC and reducing the incidence of clinical mastitis. However, the above supplement had no effect on milk composition [38]. In a study by Saba et al. [39], organic and inorganic Se supplements administered to ewes induced a minor increase in the fat content and the total dry matter content of milk relative to the control group. In the present experiment, Se supplementation also increased the fat content of milk, which indicates that the synthesis and secretion of lipids from the mammary gland were not compromised in dams with lower SCC (group E). Higher SCC in milk intensify lipolytic processes by promoting the release of intracellular enzymes, lipases, and proteases. The changes in the milk fat globule membrane induced by lipase (produced by leukocytes) and plasmin (through lipoprotein hydrolysis) increase the content of free fatty acids (FFAs) in milk [40].

### 4.3. Blood Parameters

There is a general scarcity of published studies on the influence of long-acting Se preparations on hematological, biochemical, and immunological parameters in sheep. Only minor changes in the above parameters were noted in the present study. Other researchers reported similar results in calves and correlated these changes with age and physiological development [41].

In this experiment, the significant increase in serum total protein in supplemented lambs probably resulted from an improvement in their Se status. Selenium is responsible for protein biosynthesis, and its deficiency can lead to hypoproteinemia in ruminants [42]. The beneficial effects of Se on protein transport and biosynthesis processes were described by El-Shahat and Abdel Monem [43] who reported an increase in serum albumin and total protein levels in lambs whose dams received Se and vitamin E supplements during pregnancy.

In the current study, Se supplementation did not affect glucose levels in lambs, and similar observations were made by Abdel-Raheem et al. [44]. Triglyceride concentrations increased in control group lambs in the last stage of the experiment. Similar changes were reported by Sobiech [45] in a study of goat kids with nutritional muscular dystrophy (NMD) caused by selenium deficiency. In the present study, the significant increase in triacylglycerol levels in control group lambs could suggest that lipolysis was intensified due to Se deficiency.

Cholesterol concentration was somewhat higher in the experimental lambs, which can probably be attributed to Se supplementation. Such an effect was also noted by Abdel-Raheem et al. [44] in ewes supplemented with Se. Lower serum cholesterol in control group lambs suggests that Se deficiency could impair liver function and cholesterol synthesis.

The activities of AST and GGT were similar in all groups during the experiment, and remained within the reference ranges for lambs [46]. It should be noted that AST and GGT have the highest affinity for liver tissue; therefore, the lack of significant differences in their activities between groups points to the absence of pathological changes in the liver. The activity of LDH also remained fairly stable during the experiment. This parameter should be analyzed in young ruminants which are prone to Se deficiency and, consequently, NMD [46]. Based on the results of this study, pathological changes in muscle tissues were ruled out in all animals.

Minor fluctuations in ALP activity were observed during the study, but the noted differences were not statistically significant. The observed variations were associated with physiological maturation as well as ossification processes where ALP plays an important role [47].

The results of the present study indicate that the Se preparation enhanced defense mechanisms in lambs by increasing lysozyme activity, ceruloplasmin activity, gamma globulin levels, RBA and PKA of phagocytes, and the proliferation of mitogen-stimulated lymphocytes (MTT-ConA and MTT-LPS). As a result, the analyzed preparation enhanced both humoral and cellular immunity in lambs. The beneficial effects of Se on the immune system have also been described by other authors [23,48]. Selenium participates in immune processes, and Se compounds enhance humoral immunity and increase the concentration of immunoglobulin M [49]. Dietary Se supplements increase antibody production, enhance the phagocytic activity of neutrophils and macrophages, and increase T cell counts after mitogen stimulation [50]. Rock et al. [48] demonstrated that prenatal Se supplementation in ewes improved IgG absorption from the small intestine in lambs; therefore, the offspring of control dams were characterized by lower serum IgG levels that the offspring of experimental dams administered Se supplements during pregnancy. The same authors also found that prenatal Se supplementation increased serum IgG levels in calves. These findings indicate that Se supplements can influence IgM synthesis in dams and IgG absorption in newborns.

### 4.4. Lamb’s Growth Rate and Indicators of Musculature and Fatness

The analyzed Se supplement stimulated the growth rate and muscle development in lambs without affecting carcass fatness. These effects were more pronounced in lambs that were directly injected with barium selenate. These lambs were characterized by larger cross-sectional area of m.l.d. and higher meatiness [21]. It should be stressed that the above changes were not accompanied by an increase in fatness, as demonstrated by similar fat thickness over the loin eye. The growth rate was somewhat higher in the offspring of dams receiving the Se preparation, which could be attributed to the influence of Se on the chemical composition of milk that was more abundant in fat and, consequently, dry matter. The results of studies investigating the growth-promoting effects of Se supplements in lambs are inconclusive. These discrepancies could be attributed to differences in the administered forms of supplemental Se, different dietary levels of Se, and interactions with other chemical compounds. According to many authors, Se combined with vitamin E improved ADG and feed conversion efficiency [51,52] in lambs, whereas Se administered with yeast improved growth performance in lambs [53] and growing male goats [54]. In a study by Mohri et al. [55], Se and vitamin E supplements did not affect the final BW or ADG of lambs. Alimohamady et al. [56], Sushma et al. [57], and Vignola et al. [58] found that Se supplementation did not influence growth performance in lambs. Selenium also did not considerably affect the growth rate of buffalos [59], or ADG, feed intake, and feed conversion in goats [60]. In contrast, Kumar et al. [61] and Ibrahim and Mohamed [62] reported higher ADG in sheep whose diets were supplemented with various sources of Se. Kumar et al. [63] observed a higher growth rate in lambs receiving Se supplements (0.15 or 0.3 ppm), but significant differences were not reported between groups administered different doses of Se. Dietary Se supplementation significantly increased BW gain in goat kids: The BW determined in experimental group kids at 5 months of age was achieved by control group kids at 7.5 months of age [64].

The effects of Se preparations administered to ewes on the growth rate of lambs were analyzed by many authors. Gabryszuk and Klewiec [65] found that Se injections administered to dams before mating enhanced the growth performance of their offspring. The above results are consistent with the findings of Muñoz et al. [22] who observed that the offspring of dams administered barium selenate were characterized by similar birth weight, but significantly higher ADG during rearing than control group lambs. Zarbalizadeh-Saed et al. [66] and Abdel-Raheem et al. [44] also reported a significantly higher growth rate of the offspring of dams whose diets were supplemented with Se. Similar results were noted by El-Shahat and Abdel Monem [44] and Soliman et al. [67] who injected dams with Se and vitamin E, and Saba et al. [39] who supplemented ewe diets with Se yeast. The mechanism responsible for the growth-promoting effects of Se and vitamin E has not been fully elucidated to date. Pisek et al. [68] suggested that Se supplements could promote growth by enhancing the activity of thyroid hormones. The growth-promoting effects of Se supplements could also be attributed to the antioxidant properties of glutathione peroxidase (GPx), which is a Se-containing enzyme [44].

## 5. Conclusions

The results of the present study support the formulation of the following conclusions:

1. Barium selenate significantly improved the Se status of lambs, regardless of whether it was administered to pregnant ewes (E group) or directly to lambs in the first week of their life (EI group).
2. The milk of ewes receiving the Se supplement was characterized by significantly higher fat content and, consequently, higher dry matter concentration.
3. The analyzed Se preparation induced significant changes in immunological parameters, thus enhancing defense mechanisms in lambs.
4. The preparation in the form of barium selenate (VI) administered to lambs from the EI group had a more stimulating effect on their humoral and cellular immune response than in the lambs from the E group, where the preparation was administered to their mothers.

The results of this study indicate that the long-acting Se preparation delivers benefits to sheep by enhancing their immunity and, therefore, improving productivity.

## Figures and Tables

**Table 1:** Chemical composition of dams diets (% of fresh matter).

Specification	CJ^®^ Concentrate	Cereal Straw	Meadow Hay	Dried Beet Pulp
**Chemical Composition**				
Dry matter (%)	88.99	91.01	84.98	93.11
Crude ash (%)	5.59	7.01	9.79	4.81
Crude protein (%)	19.32	4.22	7.25	5.05
Crude fat (%)	3.42	1.54	0.82	0.82
Crude fiber (%)	6.92	43.2	28.62	14.03
Selenium (mg/kg)	0.15	0.025	0.023	0.047
Gross energy MJ kg^−1^	16.14	16.89	16.22	15.88

**Table 2:** Daily milk yield, milk composition, somatic cell counts, and selenium concentration in milk.

Parameter	Lactation Day 28			Lactation Day 70			SEM	*p*-Value		
	C (n = 10)	E (n = 10)	EI (n = 10)	C (n = 10)	E (n = 10)	EI (n = 10)		Group	Date	Group × Date
Milk yield (mL/d)	1528.4	1567.8	1537.3	1056.2	1083.6	1047.7	32.97	0.053	0.095	0.748
Dry matter (%)	3.82	16.36	15.66	15.82 ^b^	16.72 ^a^	16.12	1.096	0.021	0.110	0.994
Fat (%)	5.05	5.67	5.27	5.29 ^b^	6.02 ^a^	5.64	0.620	0.056	0.151	0.969
Protein (%)	4.72	5.12	4.80	4.95	5.28	4.91	0.174	0.075	0.321	0.750
Lactose (%)	5.23	5.26	5.29	5.28	5.35	5.26	0.061	0.835	0.582	0.743
SCC (10^3^/^mL^)	272.00	201.50	226.00	140.10	134.50	150.40	12.97	0.363	0.111	0.584
Selenium (μg/L)	0.134	0.164	0.121	0.245	0.325	0.241	0.001	0.542	0.058	0.366

a, b—*p* ≤ 0.05; SCC—somatic cell count; SEM—standard error of measurement.

**Table 3:** Hematological parameters in lambs.

Parameter	4–7 Days of Age (Day 0)			28 Day of Age			70 Day of Age			100 Day of Age			SEM	*p*-Value		
	C (n = 12)	E (n = 12)	EI (n = 12)	C (n = 12)	E (n = 12)	EI (n = 12)	C (n = 12)	E (n = 12)	EI (n = 12)	C (n = 12)	E (n = 12)	EI (n = 12)		Group	Date	Group × Date
WBC(10^9^/L)	8.38	8.42	8.41	8.43	8.46	8.83	8.44	8.52	9.17	8.41	8.59	9.79	4.06	0.132	0.095	0.167
RBC(10^12^/L)	10.28	10.33	10.32	10.38	10.45	10.59	10.39	10.42	10.92	10.37	10.48	11.10	0.958	0.280	0.169	0.106
HGB (g/dL)	11.28	11.33	11.35	11.33	11.51	11.63	11.29	11.67	12.04	11.28	11.70	12.01	0.622	0.101	0.194	0.652
HCT (%)	34.83	34.87	34.79	35.12	35.16	35.11	35.02	35.36	35.28	35.25	35.62	35.53	4.83	0.521	0.156	0.247
MCV (fl)	34.97	34.88	34.92	35.83	34.50	33.84	35.92	34.41	33.58	34.91	34.02	32.36	4.98	0.332	0.920	0. 496
MCH (pg)	10.28	10.27	10.29	10.33	10.29	10.29	10.32	10.30	10.29	10.33	10.29	10.27	0.598	0.277	0.068	0.428
MCHC(g/dL)	31.98	32.51	32.84	32.51	32.60	33.06	32.94	33.21	33.18	33.12	33.44	33.51	1.64	0.077	0.376	0.204
PLT (10^9^/L)	653.71	633.12	663.78	663.12	648.45	689.82	689.33	673.19	708.22	673.24	685.91	699.14	24.70	0.780	0.158	0.093
MPV (fl)	8.27	8.46	8.12	9.29	9.45	9.02	9.34	9.51	9.38	9.02	9.41	9.44	0.797	0.135	0.112	0.289

SEM—standard error of measurement, WBC—white blood cell counts, RBC—red blood cell counts, HBG—hemoglobin, HCT—hematocrit, MCV—mean corpuscular volume, MCH—mean corpuscular hemoglobin, MCHC—mean corpuscular hemoglobin concentration, PLT—platelet count, MPV—mean platelet volume.

**Table 4:** Biochemical parameters in lambs.

Parameter	4–7 Days of Age (Day 0)			28 Day of Age			70 Day Of Age			100 Day of Age			SEM	*p*-Value		
	C (n = 12)	E (n = 12)	EI (n = 12)	C (n = 12)	E (n = 12)	EI (n = 12)	C (n = 12)	E (n = 12)	EI (n = 12)	C (n = 12)	E (n = 12)	EI (n = 12)		Group	Date	Group× Date
GLU (mmol/L)	4.75	4.80	4.79	4.76	4.82	4.83	4.72	4.81	4.84	4.74	4.83	4.89	0.170	0.090	0.900	0.119
TP (g/L)	48.22	57.34	48.21	47.12 ^B^	60.45 ^B^	69.87 ^A^**	48.91 ^B^	55.33 ^B^	67.37 ^A^	48.03 ^B^	47.19 ^B^	68.45 ^A^*	29.6	0.002	0.002	0.694
AST (U/L)	75.04	64.16	74.08	82.96	64.22	64.18	87.28	64.21	64.02	90.59	64.34	64.12	36.70	0.057	0.101	0.114
ALP (U/L)	325.64	338.92	329.55	350.87	365.39	352.64	399.25	372.44	392.39	380.72	398.58	402.56	115.86	0.657	0.218	0.157
LDH (U/L)	1040.00	938.54	1002.71	1187.37	962.56	972.59	1222.45	973.92	994.13	1253.37	983.15	997.98	229.19	0.714	0.140	0.615
GGT (U/L)	56.82	57.34	57.92	57.88	57.45	58.02	59.32	60.02	59.89	59.98	60.12	60.08	11.88	0.716	0.178	0.229
Chol (mmol/L)	2.02	2.15	2.14	2.09	2.20	2.22	2.07	2.25	2.28	2.09	2.23	2.28	0.679	0.323	0.145	0.515
TG (mmol/L)	0.42	0.36	0.38	0.45	0.37	0.37	0.47	0.37	0.36	0.52 ^a^	0.39 ^b^	0.38 ^b^	0.030	0.017	0.169	0.761

a, b—*p* ≤ 0.05; A, B—*p* ≤ 0.01. * *p* ≤ 0.05; ** *p* ≤ 0.01—significant difference relative to day 0. SEM—standard error of measurement. GLU—glucose, TP—total protein, AST—the activity of aspartate transaminase, ALP—the activity of alkaline phosphatase, LDH—lactate dehydrogenase, GGT—gamma-glutamyl transpeptidase, Chol—the concentrations of cholesterol, TG—triglycerides.

**Table 5:** Immunological parameters in lambs.

Parameter	4–7 Days of Age (Day 0)			28 Day of Age			70 Day of Age			100 Day of Age			SEM	*p*-Value		
	C (n = 12)	E (n = 12)	EI (n = 12)	C (n = 12)	E (n = 12)	EI (n = 12)	C (n = 12)	E (n = 12)	EI (n = 12)	C (n = 12)	E (n = 12)	EI (n = 12)		Group	Time	Group× Time
Lysozyme (mg/L)	0.92	0.94	1.02	0.92 ^b^	0.94 ^b^	1.05 ^a^	0.86 ^B^	0.98 ^B^	1.12 ^A^**	0.90 ^B^	1.02 ^B^*	1.16 ^A^**	0.022	0.001	0.001	0.228
Ceruloplasmin (U/L)	51.55	51.89	52.01	50.30 ^b^	51.82 ^b^	53.09 ^a^*	52.26 ^B^	52.02 ^B^	56.81 ^A^**	51.27 ^B^	52.45 ^B^	56.23 ^A^**	9.00	0.004	0.002	0.890
Gamma globulin (g/L)	26.62	26.23	26.15	23.80 ^b^	26.89 ^b^	32.87 ^a^*	23.90 ^B^	26.54 ^B^	35.06 ^A^**	23.55 ^B^	26.38 ^B^	35.81 ^A^**	5.33	0.004	0.001	0.082
RBA (OD 620 nm)	0.52	0.53	0.63	0.49	0.53	0.59	0.48 ^B^	0.56 ^B^	0.61 ^A^	0.51 ^B^	0.58 ^B^*	0.63 ^A^	0.074	0.007	0.614	0.720
PKA (OD 620 nm)	0.39	0.40	0.43	0.37 ^b^	0.45 ^b^*	0.48 ^a^	0.37 ^b^	0.44 ^b^	0.49 ^a^*	0.35 ^B^	0.46 ^B^*	0.52 ^A^**	0.013	0.004	0.001	0.350
MTT-ConA (RI)	1.11	1.09	1.09	1.11 ^b^	1.18 ^b^**	1.19 ^a^**	1.13	1.17 **	1.19 **	1.13 ^b^	1.20 ^b^**	1.21 ^a^**	0.205	0.034	0.002	0.839
MTT-LPS (RI)	1.02	1.03	1.08	0.94 ^b^	1.10 ^b^	1.16 ^a^*	0.90 ^B^*	1.12 ^B^*	1.28 ^A^**	0.98	1.08	1.15	0.055	0.012	0.049	0.080

a, b—*p* ≤ 0.05; A, B—*p* ≤ 0.01; * *p* ≤ 0.05; ** *p* ≤ 0.01—significant difference relative to day 0; SEM—standard error of measurement. RBA—respiratory burst activity, PKA—potential killing activity, MTT-ConA—Proliferative response of T-cells stimulated by mitogen concavalin A, MTT-LPS -Proliferative response of B-cells stimulated by mitogen lipopolysaccharide.

**Table 6:** Body weights, average daily gain, and the results of ultrasonic measurements performed in lambs.

Parameter	Group			SEM	*p*-Value
	C (n = 12)	E (n = 12)	EI (n = 12)		
Initial body weight (kg)—2 days of age	5.24	5.28	5.31	0.654	0.980
Average body weight (kg)—4–7 days of age	6.08	6.16	6.26	0.698	0.916
Final body weight (kg)—100 days of age	28.44 ^b^	29.57	31.47 ^a^	12.09	0.011
Average daily gain—ADG (g)	239.12 ^b^	250.34	269.71 ^a^	101.10	0.073
m.l.d. at 100 days of age:					
height (cm)	2.18 ^B^	2.26	2.38 ^A^	0.048	0.007
width (cm)	5.63 ^Bb^	6.14 ^a^	6.31 ^A^	0.228	0.005
cross-sectional area (cm^2^)	8.63 ^Bb^	10.04 ^a^	10.28 ^A^	1.68	0.009
Fat thickness over the loin eye at 100 days of age (cm)	0.21	0.25	0.21	0.003	0.149

a, b—*p* ≤ 0.05; A, B—*p* ≤ 0.01. m.l.d.—*musculus longissimus dorsi*.

## Data Availability

Not applicable.
